# Novel Surrogate Indicators of Intracranial Meningioma Consistency and Outcomes After Resection

**DOI:** 10.1227/ons.0000000000001902

**Published:** 2026-01-15

**Authors:** Yash Akkara, Joshua J. Hon, Yesh Meadipudi, Alisha Mahmud, Maaira Khan, Ramesh Nair, Joe M. Das

**Affiliations:** 1 Imperial College School of Medicine, London, UK;; 2 Imperial College Healthcare NHS Trust, London, UK

**Keywords:** Meningioma, Consistency, Signal intensity, Psammoma bodies, Surgery

## Abstract

**BACKGROUND AND OBJECTIVES:**

Tumor consistency affects resection and outcomes in meningioma surgery. This study aimed to identify predictors of tumor consistency using the Zada grading scale and assess their association with surgical outcomes and prognosis.

**METHODS:**

We retrospectively reviewed patients aged 18 years and older who underwent intracranial meningioma resection from 2014 to 2024, with ≥1 year of follow-up. Tumors were graded into 5 consistency types per Zada's criteria. Data on demographics, imaging (T2-weighted MRI), histology, and surgical outcomes were collected. Signal intensity ratios between tumor and cortex were calculated. Statistical analyses included analysis of variance, linear regression, receiver operating characteristic analysis, and Cox regression for progression-free survival (PFS).

**RESULTS:**

Five hundred and fifty-four patients (67.7% female; mean age 58.1) were included. Firmer tumors were associated with longer operative times (492 vs 382.3 minutes for type 4 vs type 2, *P* < .0001), higher subtotal resection rates (58.6% vs 15%, *P* < .0001), and increased psammoma body prevalence (17.2% vs 3%, *P* < .0001). Adherence to surrounding structures correlated with firmness (β = 0.2953, *P* = .0046). Softer tumors showed higher SI ratios (1.2 vs 0.95, *P* = .0027), with an area under the curve value of 0.6888. SI thresholds <0.6533 and >1.547 predicted firm and soft tumors with 95% sensitivity respectively. Type 2 tumors had the lowest complication rates (1.56%), whereas type 5 had the highest reoperation rates (33.3%). Firmer tumors were linked to worse PFS (*P* = .0247), primarily due to lower resection extent (β = −0.8752, *P* = .0007).

**CONCLUSION:**

Higher meningioma consistency is linked to surgical difficulty and poorer PFS. Psammoma bodies, tissue adherence, and SI ratios are promising surrogate markers to guide surgical planning.

**ABBREVIATIONS:**
EMR: electronic medical record
EOR: extent of resection
KPS: Karnofsky Performance Status
SI: signal-intensity.

Meningiomas are common benign intracranial neoplasms,^1^ accounting for 37.6% of brain tumors.^2^ They are more frequent in women, with incidence increasing with age.^3^ Meningioma consistency is a useful factor in operative planning, with scope to directly affect surgical approach and extent-of-resection (EOR). Tumor consistency refers to the physical properties of a tumor, such as firmness and pliability. In 2013, Zada et al^4^ proposed a scoring scale to standardize and objectively grade meningioma consistency. The grading system involves 3 parameters, each assigned a consistency type from 1 to 5 including general descriptions of the physical texture of the tumor (ranging from extremely soft to extremely firm/calcified), capsule characteristics (ranging from easily folded to rigid and unfoldable), and instruments used for internal debulking (ranging from suction to cautery loop/ultrasonic aspirations).

Current research has briefly explored the links between meningioma consistency on various metrics, including patient demographics, symptoms, histopathological characteristics, MRI features, EOR, and postoperative complications,^5^ with findings suggesting tumor consistency may serve as a predictive factor for surgical outcomes. On the other hand, few advances have been made to the grading system proposed by Zada et al since 2013, with a lack of studies exploring the scale's accuracy at representing true consistency. As such, this highlights the need for more quantitative markers, such as genetic or imaging biomarkers, to ensure reproducibility and accurate comparisons. This study aimed to comprehensively identify and validate the surrogate indicators of meningioma consistency, using Zada's standardized grading system, and assess their impact on surgical parameters, EOR, and postoperative outcomes.

## METHODS

### Study Design

This is a retrospective cohort study of all patients treated at our institution between January 2010 and January 2024 with a diagnosis of an intracranial meningioma. All patients were identified using a formal audit request on the electronic medical record (EMR) system. This study was conducted following principles outlined in the Declaration of Helsinki. This study was registered and approved as a formal audit within our institution. All included patients provided consent for their data to be used in retrospective studies on admission. This study used confidential patient data within our institution's database. Any data requests may be made to the corresponding author.

### Participants

All patients were required to have a diagnosis of an intracranial meningioma that underwent direct resection. Patients with spinal meningiomas or those with uncertain histologies were excluded from the analysis. All patients were required to have postoperative histology reports regarding the composition of tumor tissue, with at least 1 T2-weighted MRI scan preoperatively. All patients were aged 18 years or older, and had at least 1-year of follow-up after resection. Any patients with incomplete data on the EMR system or inadequate follow-up were excluded from the study.

### Outcomes

5 authors (YA, JJH, YM, AM, MK) performed retrospective data-extraction of all included patients on the EMR system, including assessing surgical notes, histology reports, and outpatient follow-up data. The following outcomes were assessed:

Baseline demographic factors (age, sex, Karnofsky Performance Status [KPS]), tumor characteristics [consistency type, size, location, histological characteristics, signal-intensity (SI) ratio], surgical characteristics (touchtime, length-of-stay, EOR, complications, reoperations, adjuvant therapy), and surgical outcomes [KPS at discharge, complications, progression-free survival (PFS)].

The SI ratio was calculated as the ratio of intensity of the meningioma vs the patient's cerebral cortex as per the protocol demonstrated by Watanabe et al.^6^ As per their protocol, circular regions of interest (ROIs) were manually placed on T2-weighted MRI sequences within the central portion of the tumor, avoiding areas of intra-ROI heterogeneity or necrosis. A comparable ROI was placed within the ipsilateral cerebral cortex (superior frontal or inferior temporal gyrus), ensuring comparable imaging depth and sequence parameters. The SI ratio was then defined as the mean tumor SI divided by the mean cortical SI, representing the relative tumor-to-cortex signal intensity (SI).^6^ Medical vs surgical complications were defined by the Landriel-Ibañez classification system.^7^

### Comparison and Grouping

Using the grading system developed by Zada et al,^4^ patients were categorized into 1 of 5 types, with the final allocation being based on overall “consistency score” criterion from the initial grading system, determined using a combination of the 3 subcriteria (general description, capsule characteristics, instruments used), as per the original protocol (**Supplementary Table**, http://links.lww.com/ONS/B282). Any tumors with heterogenous compositions or uncertainty in consistency scoring were discussed among authors until a consensus was reached. Additional factors such as psammoma bodies, vascularity, and adherence to surrounding structures were also examined. Data regarding psammoma bodies and vascularity were identified using histology reports of the tumor parenchyma, with adherence to surrounding structures identified using intraoperative reports.

### Statistical Analysis

The Shapiro-Wilk Test, *t* test, and analysis of variance were used to determine statistically significant differences in baseline and postoperative characteristics across patient groups. The SI for both meningiomas and the cerebral cortex were calculated using the *Philips IntelliSpace PACS 4.4* system (Philips Healthcare SpA), with intensities compared to obtain the ratio. Receiver-operating characteristic (ROC) curves were produced using the SI ratio, with patients with softer tumors (type 1-2) considered as “negatives”, and harder tumors (type 3-5) considered as “positives”. Kaplan-Meier analyses were performed to compare trajectories of complication profiles and PFS of patients, with Cox-proportional hazards regression used to identify factors driving potential differences in complications and/or PFS.

## RESULTS

A total of 1391 patients underwent resections for meningiomas at our institution between January 2014 and January 2024. Within this subset of patients, 481 did not have sufficient follow-up and clinical data on the EMR system, whereas an additional 356 had tumor consistencies that were unable to be resolved on chart review (due to a lack of description in operative notes or inconclusive imaging), due to differences in standards of reporting over time.

As such, 554 patients (179 M, 375 F) made up the final sample of included patients, with a mean age of 58.13 (15.12) years. Of these patients, 60, 128, 296, 58, and 12 patients scored 1, 2, 3, 4, and 5, respectively, on the meningioma consistency grading scale. Median (±IQR) follow-up across patients was 55 (27-78) months. Overall and consistency-specific patient demographic characteristics are described in Table 1.

**TABLE 1.** Baseline Demographic Characteristics of Participants

Demographics	Overall (n = 554)	Consistency type				
		1 (n = 60)	2 (n = 128)	3 (n = 296)	4 (n = 58)	5 (n = 12)
Sex						
Male	179	20	44	98	14	3
Female	375	40	84	198	44	9
Age	58.13 (15.12)	55.16 (13.14)	58.32 (11.32)	59.24 (16.73)	54.45 (14.32)	61.63 (15.40)
Deprivation	5 (3-7)	5 (4-6.5)	5 (3-6)	5 (4-7)	5 (3-6)	5 (4-6)
Location						
Skull-base	182	21	46	92	18	5
Convexity	124	12	31	59	18	4
Frontal	80	5	19	53	3	0
Parasagittal	72	7	15	40	8	2
Temporal	44	3	10	26	5	0
Tentorial	7	2	0	4	1	0
Other	45	10	7	22	5	1
Follow-up (median, IQR, mo)	55 (27-78)	65 (39-94)	55 (38-78)	50 (25-67)	45 (24-66)	48 (35-60)

A variety of tumor and surgical characteristics were compared across the 5 groups. Consistency type 1 meningiomas reported a significantly lower mean size vs other groups, particularly type 3 (38.40 vs 48.35, *P* = .0007) and 4 (38.40 vs 50.58, *P* = .0014) meningiomas. Type 1 meningiomas were also significantly more likely to be grade 1 meningiomas vs type 2 meningiomas (*P* = .0173), with no other significant differences in tumor grade identified. A substantial trend was observed with higher meningioma consistency associated with a longer duration of surgery (*P* < .0001). Type 1 meningiomas reported the lowest median duration (300.6), followed by type 2 (382.3 minutes), type 3 (427.4 minutes), and type 4 (492.0). A notable difference was observed in the frequency of subtotal resection across groups (*P* < .0001), with type 1 meningiomas reporting the lowest rate of subtotal resection (15%), followed by type 2 (35.9%), type 3 (44.9%), type 4 (58.6%), and type 5 (66.7%). No significant differences were found in preoperative KPS or length-of-stay after resection across the groups. These findings are described in Figure 1.

**FIGURE 1.**
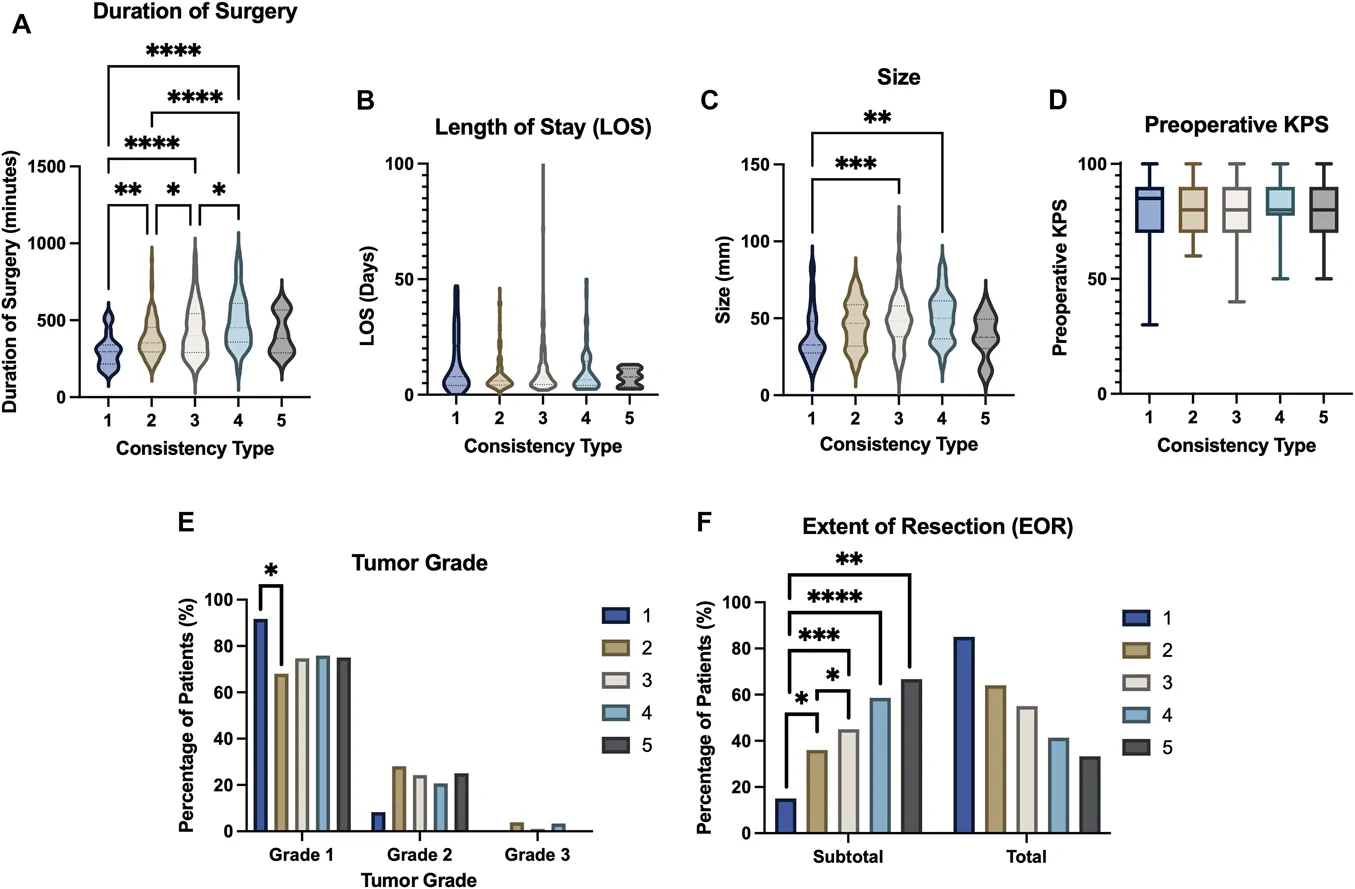
Differences in surgical duration (**A**), LOS (**B**), tumor size (**C**), preoperative KPS (**D**), tumor grade (**E**), and EOR (**F**) across consistency types. EOR, extent of resection; KPS, Karnofsky Performance Status; LOS, length-of-stay.

### Novel Indicators

We examined 5 additional indicators to identify their role in meningioma consistency: vascularity, adherence to surrounding structures, collagen content, psammoma bodies, and SI ratio on imaging. Type 1 meningiomas were found to have significantly fewer instances of highly-adhered tumors vs type 3 meningiomas (26.7% vs 58.1%, *P* < .05), with a generally increasing (but insignificant) trend identified between increased consistency and surrounding adhesion. Interestingly, we found type 4 meningiomas showed a significantly higher frequency of psammoma bodies on histology (17.2%) vs type 3 (6.1%, *P* < .001), type 2 (3%, *P* < .0001), and type 1 (0%, *P* < .0001) meningiomas, suggesting an increasing presence as consistency increases. On the other hand, we found no significant differences across consistency types regarding vascularity and collagen content on histology. In addition, we performed multiple linear regressions between consistency type and vascularity, adherence, collagen content, and psammoma bodies (Table 2). Both adherence to surrounding structures (β = 0.2953, *P* = .0046) and the presence of psammoma bodies (β = 0.7175, *P* = .0003) were found to be significantly linked with meningioma consistency (Figure 2A).

**TABLE 2.** Multiple Linear Regressions of Predictors of Meningioma Consistency

Variable	β	95% CI (asymptotic)	*P*
Intercept	2.462	2.300 to 2.624	**<.0001**
Vascularity	0.02206	−0.1964 to 0.2405	.8425
Adherence to surrounding structures	0.2953	0.09205 to 0.4985	**.0046**
Collagen content	0.3968	−0.09944 to 0.8930	.1166
Psammoma bodies	0.7175	0.3352 to 1.100	**.0003**

Bold indicates statistical significance (*P* < .05).

**FIGURE 2.**
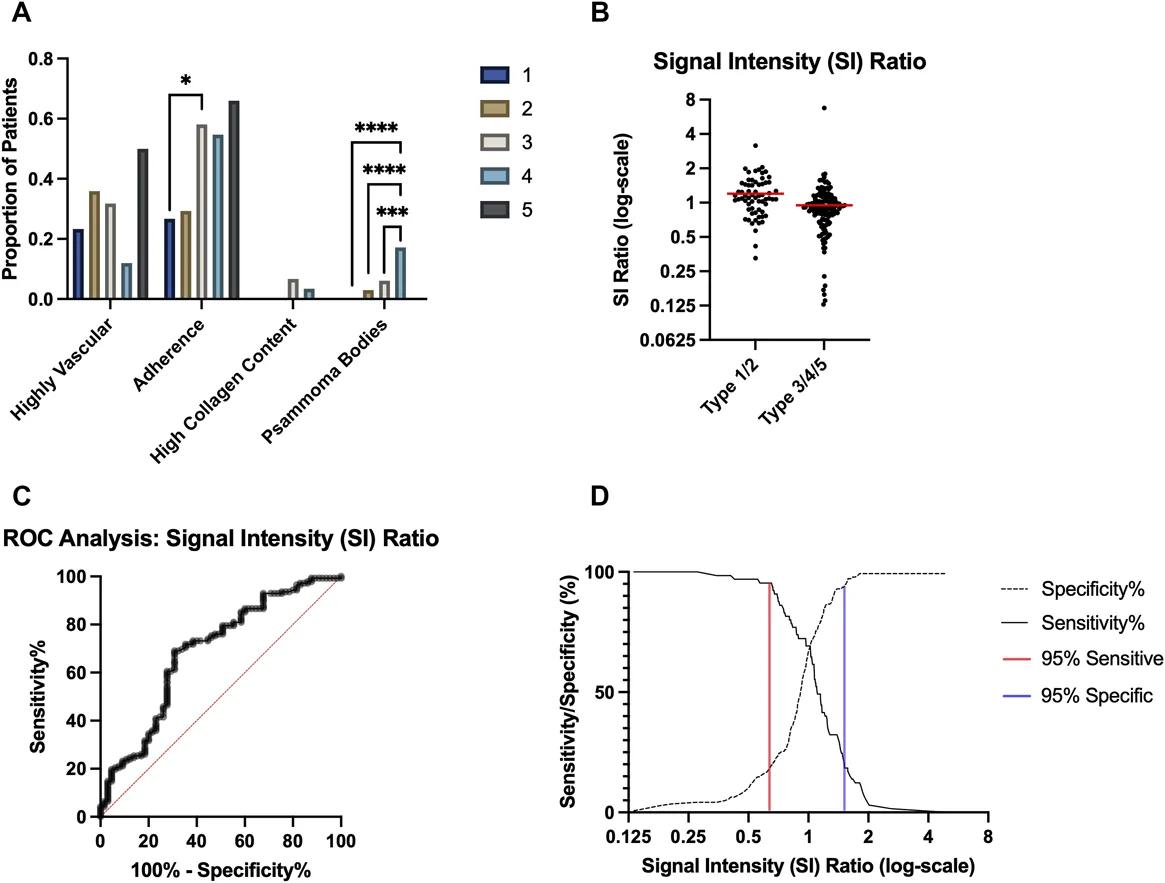
Differences in histological characteristics (**A**) and SI ratio across consistency types (**B**), with ROC (**C**) and sensitivity/specificity (**D**) curves for the SI ratio. ROC, receiver operating characteristic; SI, signal intensity.

We also performed an analysis on the SI of the tumor compared with the patient's cerebral cortex, deriving the SI ratio. The SI ratio was found to be significantly higher in softer tumors (type 1/2) compared with harder tumors (type 3-5) (1.2 vs 0.95, *P* = .0027). ROC analysis (considering softer tumors as negatives and harder tumors as positives) using SI ratio differences revealed an area under the curve of 0.6888 (95% CI [0.6082, 0.7695], *P* < .0001). SI ratio cutoffs of <0.6533 and >1.547 were 95% sensitive and 95% specific, respectively. These findings are described in Figure 2B-D.

### Outcomes and Prognostic Role of Consistency

We compared a variety of surgical outcomes with identify the prognostic role of consistency longitudinally (Figure 3A). Type 2 meningiomas in particular were associated with a significantly lower risk of medical complications during inpatient stays (1.56%) vs both type 3 (12.16%, *P* < .05) and type 4 (17.24%, *P* < .05) meningiomas. An increasing trend was observed with an increasing frequency of reoperations with increased consistency. Type 5 meningiomas reported the highest rate of reoperations (33.3%), followed by type 4 (20.6%), type 3 (13.5%), and type 2 (10.9%) meningiomas (*P* < .01). No significant differences were observed in the rate of surgical complications or postoperative KPS across the 5 groups (Figure 3B).

**FIGURE 3.**
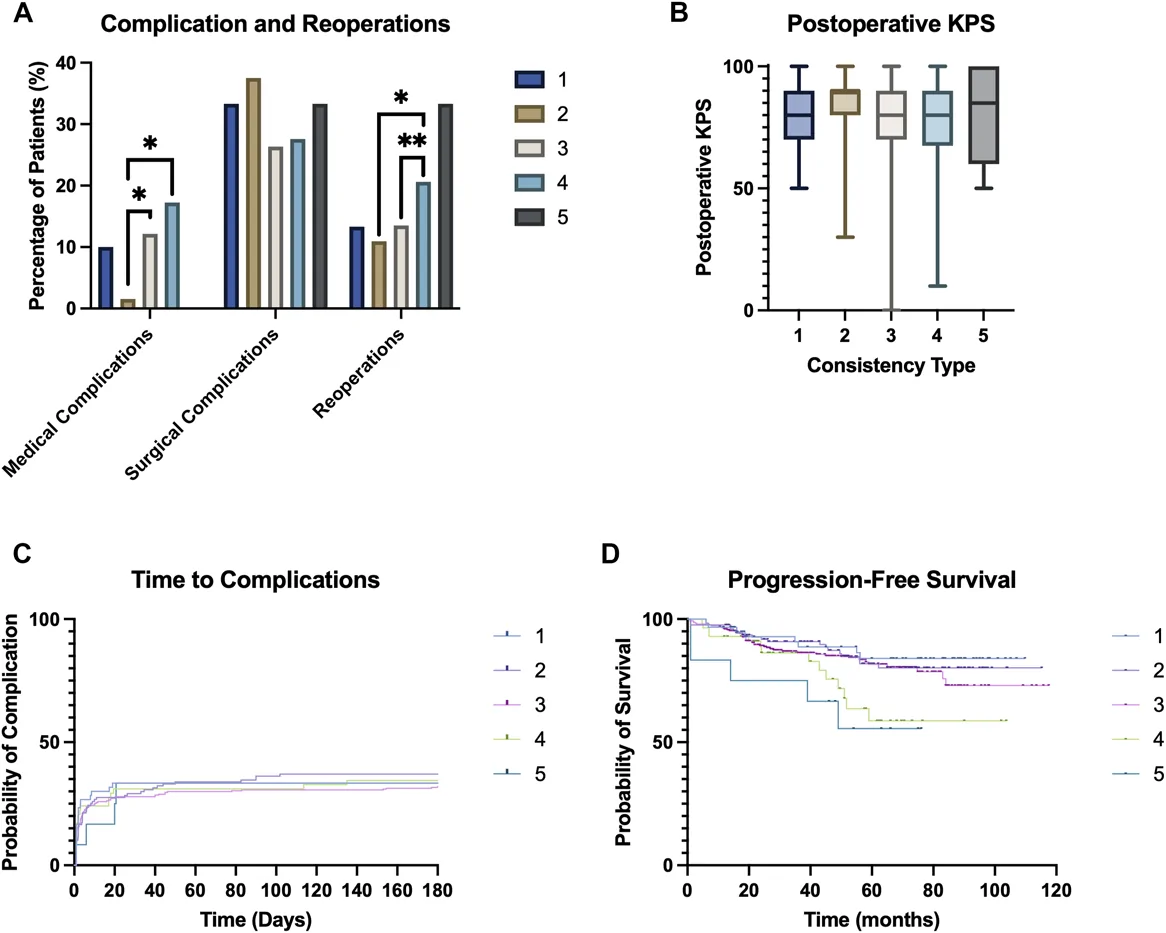
Differences in complications/reoperations (**A**), postoperative KPS (**B**), time-to-complications (**C**), and progression-free survival (**D**) across consistency types. KPS, Karnofsky Performance Status.

Kaplan-Meier analysis revealed no significant differences in the trajectory of complications across the 5 groups, with generally similar complication profiles at 6 months after surgery (Figure 3C). Longitudinal analysis of PFS revealed significant differences across the groups (Figure 3D), with type 4 and 5 meningiomas being significantly more likely to report worse PFS vs type 1, 2, and 3 meningiomas (*P* = .0247). Cox proportional hazards regression revealed the driving factor behind decreased PFS in type 4 and 5 meningioma as reduced EOR in harder tumors (β = −0.8752, 95% CI [−1.391, −0.3783], *P* = .0007).

## DISCUSSION

This study presents novel findings regarding surrogate indicators of meningioma consistency and their impact on surgical outcomes, potentially providing clinicians with valuable tools to anticipate tumor consistency before surgery, potentially improving surgical planning and ultimately, clinical outcomes for patients with intracranial meningiomas.

Type 1 meningiomas demonstrated a significantly lower mean size compared with firmer groups, consistent with findings by Romani et al.^8^ The finding may reflect reduced stromal and calcified components of softer meningiomas. These tumors typically exhibit higher water content and fewer fibrous or mineralized regions, causing reduced intrinsic rigidity and structural density.^9,10^ As such, this may allow for greater deformability and less volumetric expansion compared with firmer, more calcified tumors, potentially resulting in smaller-appearing tumors on imaging or intraoperatively.^11,12^ Regarding histopathological grade, consistency type 1 meningiomas were significantly more likely to be World Health Organization grade 1 compared with consistency type 2 meningiomas. This association may reflect the lower mitotic rate (<4/10 high power fields) of grade 1 meningiomas, which likely contributes to softer consistencies.^1^

Higher meningioma consistency was significantly associated with increased operative duration, consistent with the findings of Itamura et al.^13^ This is likely due to greater adherence to surrounding structures, requiring careful piecemeal approaches to resection. Lower meningioma consistency was also associated with a lower rate of subtotal resection, consistent with the findings of Al Abdulsalam et al^14^ and Itamura et al.^13^ This association is primarily explained by the greater adherence to surrounding structures observed in firmer meningiomas, often necessitating leaving residual tumor to preserve neurological function.

Our multivariate analysis revealed that both adherence to surrounding structures and psammoma bodies were significantly linked with firmness. The increase in adherence across consistency types may reflect differences in tumor biology and growth patterns.^15^ On the other hand, this association may also be influenced by the integrity of the arachnoid interface. Recent work has highlighted the critical role of the arachnoid membrane in defining the tumor–brain interface.^16^ The absence or fragmentation of the arachnoidal plane may therefore reflect not only mechanical firmness but also underlying biological invasiveness, consistent with reports that more aggressive tumors tend to erode the arachnoid and invade adjacent cisterns.^16^ Thus, the observed relationship between consistency and adherence in our cohort is likely multifactorial, reflecting both the physical properties of the tumor and the biological integrity of the arachnoid barrier. This association between consistency and adherence has important surgical implications, as extensive adherence contributes to subtotal resection.^17^ Psammoma bodies were also significantly more frequent in type 4 meningiomas compared with softer consistency meningiomas.^18,19^ The progressive increase in calcification density correlates with increased hardness, as previously suggested by Itamura et al^13^ and Nagao et al,^20^ serving as a potential predictor of future consistency in cases of reoperations.

The SI ratio emerged as a promising preoperative predictor of meningioma consistency. We found that softer tumors demonstrated significantly higher SI ratios, aligning with the fundamental principles of MRI, where tissues with higher water content (characteristic of softer meningiomas) exhibit hyperintensity on T2-weighted sequences.^10,21-23^ Our ROC analysis revealed a moderate diagnostic accuracy for the SI ratio as a predictor of consistency. Particularly noteworthy were the identified cutoff values of <0.6533 and >1.547, being 95% sensitive and 95% specific for identifying harder tumors, respectively. Although the SI ratio has been previously examined as a marker of meningioma consistency (within previous work by Smith et al,^24^ Watanabe et al,^6^ and Han et al,^25^ who noted correlations between SI and consistency), our study expands on previous work by defining specific SI ratio thresholds corresponding to Zada's standardized consistency types, enabling preoperative grading and improving reproducibility. The clinical utility of these cutoffs is substantial, as neurosurgeons can potentially use SI ratios to anticipate tumor consistency, estimating the likelihood of gross total resection and adjusting surgical strategy. This may also enable improved counseling regarding expected outcomes and complication risks.

We found higher risks of complications and reoperations in firmer meningiomas. The impact of meningioma tumor consistency on surgical outcomes was previously explored in a cohort study of 209 patients,^5^ finding that firmer tumors were associated with lower rates of gross-total resection, higher recurrence rates, reoperations, and increased complication rates. Lower consistency tumors may be associated with reduced complication rates due to several factors: they can be suctioned with minimal manipulation of surrounding tissue, exhibit less invasiveness into adjacent structures, and generally require shorter operative durations,^26^ reducing the risk of iatrogenic injury. Higher consistency tumors are firm/fibrous, and often more invasive into surrounding nerves and blood vessels, making gross total resection difficult.^27^ Incomplete resection also increases recurrence rates, potentially explaining the higher rate of reoperations observed in our study.^28^

The significantly worse PFS observed in type 4/5 meningiomas seems to be primarily driven by the technical limitations in achieving gross total resection of these firm, highly adherent tumors, which was confirmed by our Cox-proportional hazards regression. These associations are likely explained by the higher rates of residual tumor and increased likelihood of recurrence.

We found significant value in the role of consistency in intraoperative and postoperative outcomes in meningioma resection. A notable strength of this study is the identification of quantifiable surrogate markers (adherence, psammoma bodies, and SI ratio) that may enable preoperative prediction and postoperative identification of meningioma consistency, with potential to inform future surgical strategy. Our sample size was substantial (n = 554), exceeding the sample sizes used in previous comparable literature. Using a longitudinal design facilitated an investigation of long-term outcomes after resection and how prognoses and responses to treatment evolved over time. Finally, our stratified analytical approach allowed for detailed examination of outcomes across consistency types.

### Limitations

The retrospective design introduces potential selection bias that could not be completely mitigated. Propensity score matching would have strengthened comparability between consistency types but was not implemented due to sample size constraints in the extreme consistency categories. Measurement bias in the estimation of KPS and consistency grading from retrospective clinical documentation may have occurred. Evolution in surgical techniques and technologies over the study period (2010-2024) may also have confounded outcomes measurements, although this would likely affect all consistency groups similarly. Finally, the requirement of SI ratios on T2-weighted MRI may have led to the exclusion of valuable patient data for those with inconclusive imaging, which would have otherwise strengthened our sample size.

A substantial proportion of potentially eligible patients were excluded due to insufficient follow-up or clinical data, whereas another 356 had inadequately documented consistency. This high exclusion rate may raise concerns regarding generalizability. In addition, the variation in follow-up duration (12 months-11 years) complicated interpretation of long-term outcomes. The observational nature of our study prevents the establishment of causality between consistency and outcomes. Despite showing promise, our ROC analysis of the SI ratio also obtained an area under the curve of 0.6888, demonstrating only a moderate ability to distinguish tumor consistency. Future work using more detailed imaging modalities and sequences may be beneficial in improving diagnostic accuracy. Finally, the relatively small sample sizes in the extreme consistency groups (n = 60 for type 1, n = 12 for type 5) limited statistical power for detecting significant differences involving these categories, potentially masking certain relationships. In addition, the low sample sizes of groups 1 and 5 prevented us from performing nearest-neighbor matching across all groups while preserving statistical power, preventing a higher degree of comparability across the groups. Future studies with larger samples and even distributions across consistency types would be beneficial in allowing for matching across groups.

## CONCLUSION

We found softer meningiomas were significantly smaller, displayed reduced adherence to surrounding structures, and reported a lower surgical duration and rate of subtotal resection. The presence of psammoma bodies and degree of adherence to surrounding structures were significantly associated with firmer tumors. MRI SI ratio was a promising radiological marker of consistency, allowing for greater preoperative grading prediction. Higher consistency tumors were associated with reduced EOR, increased reoperations/complications, and worse PFS. The inclusion of the MRI SI ratio (<0.65 suggesting firm consistency), tumoral adhesion patterns, and presence of psammoma bodies from previous specimens (in cases of reoperations) would enable more accurate preoperative prediction of tumor consistency, allowing surgeons to modify strategies and counsel patients about risks of surgery and recurrence.

## Supplementary Material

## Supplemental Digital Content

**Supplementary Table.** Consistency Grading System by Zada et al.^4^
