# Supplementary material for: Novel Surrogate Indicators of Intracranial Meningioma Consistency and Outcomes After Resection
Source: Oper Neurosurg. 2026 Jan 15;31(5):898–905. doi: 10.1227/ons.0000000000001902 (PMC13652121; doi:10.1227/ons.0000000000001902)
Supplement: Supplementary file 1 [file ons-31-898-s001.docx]

# Supplementary Table

***Supplementary Table 1***: Consistency Grading System by Zada et al. (2013)

| **Consistency Score/Type** | **General Description** | **Capsule Characteristics** | **Exemplary Instrument(s) Used for Internal Debulking** |
| --- | --- | --- | --- |
| 1 | extremely soft tumour | capsule easily folded or no capsule | suction |
| 2 | soft tumour; bulk of tumour freely suctioned; fibrous stroma remains | capsule easily folded following partial internal debulking | majority done w/ suction; fibrous stroma resected w/ capsule |
| 3 | average consistency; cannot be freely suctioned; requires some degree of mechanical debulking | capsule folds following sufficient debulking | piecemeal resection; ultrasonic aspiration or other mechanical debulking device, often at low setting; sharp dissection |
| 4 | firm tumour; requires mechanical debulking | firm capsule; difficult to collapse despite tumour debulking | piecemeal resection, ultrasonic aspiration, loop cautery, or other mechanical debulking, often at high setting; sharp resection or loop cautery |
| 5 | extremely firm &/or calcified tumour; may approach density of bone; often requires en bloc resection | rigid capsule that does not fold or collapse | difficult to debulk even w/ ultrasonic aspiration, cautery loop, or mechanical/sharp dissection |
